# Sickness absence in musculoskeletal disorders - patients' experiences of interactions with the Social Insurance Agency and health care. A qualitative study

**DOI:** 10.1186/1471-2458-11-107

**Published:** 2011-02-16

**Authors:** Jenny Hubertsson, Ingemar F Petersson, Barbro Arvidsson, Carina A Thorstensson

**Affiliations:** 1 Musculoskeletal Sciences, Dept of Orthopedics, Skåne University Hospital, 221 85 LUND, Clinical Sciences Lund, Lund University, Lund Sweden; 2 School of Social and Health Sciences, Halmstad University, Box 823, 301 18 Halmstad, Sweden; 3 Faculty of Health Care and Nursing Sciences, Gjøvik University College, Teknologivn. 22, 2802 Gjøvik, Norway; 4 Research and Development Centre, Spenshult, 313 92 Oskarström, Sweden

## Abstract

**Background:**

Sickness absence has represented a growing public health problem in many Western countries over the last decade. In Sweden disorders of the musculoskeletal system cause approximately one third of all sick leave. The Social Insurance Agency (SIA) and the health care system are important actors in handling the sickness absence process. The objective was to study how patients with personal experience of sickness absence due to musculoskeletal disorders perceived their contact with these actors and what they considered as obstructing or facilitating factors for recovery and return to work in this situation.

**Methods:**

In-depth interviews using open-ended questions were conducted with fifteen informants (aged 33-63, 11 women), all with experience of sickness absence due to musculoskeletal disorders and purposefully recruited to represent various backgrounds as regards diagnosis, length of sick leave and return to work. The interviews were audio-recorded, transcribed verbatim and analysed using content analysis.

**Results:**

The informants' perceived the interaction with the SIA and health care as ranging from coherent to fragmented. Being on sick leave was described as going through a process of adjustment in both private and working life. This process of adjustment was interactive and included not only the possibilities to adjust work demands and living conditions but also personal and emotional adjustment. The informants' experiences of fragmented interaction reflected a sense that their entire situation was not being taken into account. Coherent interaction was described as facilitating recovery and return to work, while fragmented interaction was described as obstructing this. The complex division of responsibilities within the Swedish rehabilitation system may hamper sickness absentees' possibilities of taking responsibility for their own rehabilitation.

**Conclusions:**

This study shows that people on sick leave considered the interaction with the SIA and health care as an important part of the rehabilitation process. The contact with these actors was perceived as affecting recovery and return to work. Working for a more coherent process of rehabilitation and offering professional guidance to patients on sick leave might have an empowering effect.

## Background

Sickness absence has represented a growing public health problem in many Western countries over the last decade [1,2]. In Sweden disorders of the musculoskeletal system cause approximately one third of all sick leave. This includes impaired working ability, long-term sick leave and disability pension [3].

In Sweden sickness benefit is provided by the Social Insurance Agency (SIA) if a disease or illness causes impaired function leading to reduced work capacity. A sick note from the physician is used as a basis for the decision made by the SIA. Also, the SIA is responsible for coordination of rehabilitative activities during sick leave and for ensuring that needs for vocational rehabilitation are met. The health care system is responsible for the medical rehabilitation of sickness absentees. The SIA and the health care system are therefore important in the processing of sickness absence decisions in Sweden [4]. The work tasks of these actors are crucial for people's security and welfare, and also in terms of the economy of the national social insurance [5,6].

At the end of the 20th and the beginning of the 21st century there was a steep rise in the number of persons on long-term sick leave in Sweden. The authorities therefore made it an important objective to bring down the numbers on sick leave, and since 2003 the amount of days with sickness cash benefit and disability pension per insured person have been decreasing. Both in the SIA and in the health care system there have been large-scale efforts and investments to accomplish these goals.

The connection between health outcomes and the context of the health care provided has been studied in various ways [7,8]. Earlier Swedish studies have concluded that the quality of the encounter between professionals in the health care system and the SIA and the person on sick leave is an important factor in the rehabilitation process that may facilitate or obstruct return to work[9-12].

The aim of our study was to enhance the understanding of how patients on sick leave due to musculoskeletal disorders perceive their interaction with different institutional actors in the rehabilitation process, and to gain a deeper understanding of these patients' priorities in this process. Our specific objectives were to study how patients with experience of sickness absence due to musculoskeletal disorders have perceived their contact with the SIA and the health care system, and what factors, in their experience, can be described as facilitating or obstructing recovery and return to work.

## Methods

In-depth interviews were used to obtain a deeper understanding of the informants' perceptions and views concerning the issue in question [13]. Fifteen patients with experiences of sickness absence due to musculoskeletal disorders were interviewed using a semi-structured interview guide with open-ended questions. The interview guide contained the following questions: 1. How have you experienced your contact with the SIA during your sick leave? 2. How have you experienced your contact with the health care system during your sick leave? 3. How have you experienced the cooperation between the health care system and SIA during your sick leave? 4. Do you have any suggestions as to how to improve the patients' situation in relation to the contact with the SIA and the health care system during sick leave?

Aspects brought up by the informants were probed in more depth, and interviews lasted between 30 and 90 minutes. To test the relevance of the interview guide two pilot interviews were conducted. No changes were made and the two interviews were included in the analysis. All interviews were conducted by an interviewer with experience of work within both the SIA and the health care system (JH). The interviews were audio-recorded and transcribed verbatim. All informants were informed before the interview about the purpose and scope of the study, and written consent was obtained.

In this study we used a tentative, explorative approach. The interviews and the analysis were not based on theories about interaction between patients/sickness absentees and other actors in the rehabilitation process. Instead, the informant's experiences and perceptions were explored in an inductive way.

People with experience of sickness absence due to musculoskeletal disorders represent a heterogeneous group, with variable background factors and characteristics affecting their experiences.

In this study we aimed to use criterion based, purposive sampling. Using such a sampling, informants are chosen because they have particular characteristics which enable detailed exploration of the phenomena at study. These may be socio-demographic characteristics or may relate to specific experiences [14].

We intended to cover a variety of predefined characteristics with respect to age, sex, residence, civil status, country of birth, sick leave status, employment status and diagnosis for sick leave. To be eligible for interview informants were required to have been on sick leave due to a musculoskeletal disorder for a minimum of six months in total over the past three years. Within this defined group we wanted to ensure diversity in informants' experiences of contact with the SIA and the health care system.

Due to patient safety and ethical reasons we could not directly approach patients or use administrative records for our sampling [14]. Instead, to ensure as broad a range as possible concerning the experiences of contact with the SIA and the health care system, informants were recruited in a two-fold way, using both purposively chosen gatekeepers and public advertisements. The gatekeepers used included 15 social insurance officers, strategically chosen to represent a geographical and organisational diversity [15], and 15 primary care physicians, randomly sampled from a list of all physicians working in primary care in the area and covering a broad geographic distribution. Furthermore, to include specialist care and patient organisations, one physician and one counselor working in the Rheumatology Department at Lund University Hospital, and a representative from the regional branch of the Swedish Rheumatism Association were also contacted. All gatekeepers were informed about the inclusion criteria and asked to approach patients and recruit them for the study.

The public advertisements were put up on notice boards at Lund University Hospital, Malmö University Hospital, and exposed by the regional Swedish Rheumatism Associations and in the local free daily paper 'Metro'.

### Data analysis

The analysis was conducted using *content analysis *[16,17]. Qualitative content analysis is a research method for subjective interpretation of the context of data through a systematic classification process of coding and identifying themes or patterns [17]. For the purpose of this study the *latent content analysis*, as described by Graneheim & Lundman, was found to be the most suitable method. Latent content analysis deals with the relationship aspect and involves an interpretation of the underlying meaning of the text, referred to as the *latent content *[16,18,19].

The analysis process started with repeated reading of the data and listening to the audiotapes to ensure accuracy during transcription, to achieve immersion and obtain a sense of the whole [17,20]. *Content areas *were constructed based on the topics of the interviews and on the specific objectives of the study. The text about the informants' experiences was then divided into *meaning units*. Each meaning unit was based on constellations of words or statements from the text that related to the same central meaning [16]. The meaning units were divided between the three content areas and the text of each content area was brought together into one text, which subsequently constituted the unit of analysis. The meaning units were *condensed*, a process of shortening the text of each unit while still preserving the core meaning, and labelled with a *code*. During the process of condensing and coding, the whole context was considered. The codes were compared and sorted into *sub-categories *constituting the manifest content. The subcategories were grouped together in *categories*. The underlying meaning in the categories was searched for and a *theme *was formulated. A theme can be understood as a thread of underlying meaning through condensed meaning units, codes, sub-categories and categories. Themes are not necessarily mutually exclusive. A condensed meaning unit, a code, a sub-category or a category can fit into more than one theme [16]. Quotes were chosen to illuminate the range of conceptions within each category.

Several steps were taken to ensure the credibility of the results [16]. The first (JH) and the last (CT) author, both with previous experience of qualitative research but with different perspectives, met regularly to discuss the process of analysis and findings. The themes and categories were also discussed with the third author (BA) and reformulated in consensus between the authors before the final labelling of the results. During the process the total context was considered by continuously going back and forth between the parts and the whole, and labels were reformulated.

### Ethical considerations

Ethical approval was obtained from the Regional Ethics Committee, Medical Faculty, Lund University (187/2007).

## Results

### Informants

The process of recruitment resulted in one informant being recruited by a social insurance officer, two by physicians in primary health care, two by the physician and one by the counsellor working in the Rheumatology Department at Lund University Hospital and one by the regional Swedish Rheumatism Association. These six informants were all included in the study. There were no responses to the advertisements put up at Lund University Hospital, Malmö University Hospital, or by the regional Swedish Rheumatism Associations. Twenty-two persons responded to the advertisement in the local free daily paper 'Metro', 15 by telephone and seven by e-mail. Of the 22 who answered the advertisement, eight were recruited consecutively. One additional informant who had sent a letter of interest after hearing about the project was recruited. Of the 15 informants chosen for participation, one did not show up at the time appointed; this was an informant recruited through the daily paper and he was replaced by another informant recruited in the same way.

Fifteen informants, aged 33-63 years, 11 women and four men, participated. Most of the informants had been on sick leave back and forth, sometimes part-time, sometimes full-time and sometimes working full-time for periods and then going back to sick leave again - this during very different lengths of time. The informant with the longest history of sick leave had been on sick leave off and on since the beginning of the nineties, and the informant with the shortest history of sick leave had been on sick leave for about six months. The fifteen informants included covered the predefined background characteristics as shown in table 2.

### Analysis

Three content areas emerged, each comprising two to four major categories. Under *Content area 1: 'Interaction with the SIA' *the categories were labelled: (a) 'tangled communication' and (b) 'uncertain subsistence'. *Content area 2: 'Interaction with the health care system' *comprised the categories (a) 'the importance of being part of an ongoing process', and (b) 'the importance of being recognized as a person'. Under *Content area 3: 'Recovery and return to work' *the categories were (a) 'possibilities to create conditions for work', (b) 'possibilities to create premises for a good life', (c) 'the link between physical and mental health', and (d) 'a wish for guidance'.

In addition, one theme that ran as a common thread through all content areas and categories was identified: 'Coherent or fragmented interaction' (Table 1).

**Table 1:** Results: Content areas, subcategories, categories and theme

Content area	Subcategories	Categories	Theme
1. Interaction with the social insurance agency	➢Getting in touch ➢The personal meeting ➢Respectful treatment ➢Communication through others	Tangled communication	
		
	➢Being questioned ➢Loss of human value ➢Being pending ➢Feeling restricted ➢Vague or incorrect information	Uncertain subsistence	
	
2. Interaction with the health care system	➢Waiting times ➢Continuity ➢Symptom relief	The importance of being part of an ongoing process	Coherent or fragmented interaction
		
	➢The personal relationship ➢Respectful treatment ➢Being understood	The importance of being recognized as a person	
	
3. Recovery and return to work	➢Trying one's ability ➢Adjusting work demands ➢Being well received	The possibilities to create conditions for work	
		
	➢Adjusting the daily routines ➢Personal preconditions ➢Fear of losing societal belonging	The possibilities to create premises for a good life	
		
	➢The link between body and soul ➢Losing self-esteem	The link between physical and mental health	
		
	➢Need for professional support ➢Being informed	A wish for guidance	

### Theme: Coherent or fragmented interaction

The informants perceived the interaction with the SIA and the health care system as spanning between coherent and fragmented. Coherent interaction was described as facilitating recovery and return to work, while fragmented interaction was described as obstructing. Coherent interaction contributed to a coherent rehabilitation process, and similarly, fragmented interaction caused a fragmented rehabilitation process.

Informants describing coherent interaction had experienced a continuous contact in which they felt like participants in an ongoing process of rehabilitation. This contact included feeling recognized as a person, being guided in the efforts to return to work, and receiving support in the personal adjustment to living with a disease. Coherent contact included both physical and psychological aspects of recovery. Informants describing fragmented interaction, on the other hand, had experienced random contact and a lack of continuity. A perceived lack of coordination led to a feeling of being alone in the process of returning to work, and depending on one's own abilities throughout the rehabilitation process.

It is important to recognize that many informants had experienced both types of interaction during their time on sick leave. The consistent implication of their experiences, however, was the description of coherent interaction as beneficial, and fragmented interaction as disadvantageous for recovery and return to work. The experiences of fragmented interaction, especially considering the contact with the social insurance office, were the most frequent, while experiences of coherent interaction were much less often described.

### Content area 1: Interaction with the SIA

In this content area three categories were identified in which the informants described the contact with the SIA as characterized by tangled communication and frustration over uncertain subsistence.

#### Tangled communication

The contact with the agency was influenced by difficulties getting in touch. The informants described the organization of the SIA as hard to grasp and the social insurance officers as hard to reach. The informants stressed the importance of personal meetings and respectful treatment, and the experiences of obtaining this varied greatly. Some informants had experienced periods of continuous, personal and respectful contact, and this was often perceived as a turning point and essential for a successful rehabilitation process. Others described the contact with the agency as random, sometimes with no contact for years and then sudden pressure and rash decisions.

But nothing happened until five, five-and-a-half years later. And then, it was all supposed to happen at 180 kilometres per hour since I had had the nerve to be on sick leave for so long. It felt so unfair. Especially as I had tried myself to do some work-based rehabilitation and so on, but never got any support. (Inf 3)

Several informants described how they often had their communication with the agency through other parties, such as social workers or lawyers, due to difficulties in the contact.

And then I asked my social worker, because I risk losing my income. And this keeps going on. One thing after the other is missing. It makes my health worse. So my social worker was kind and rang them up to ask. And then there were loads of questions. [...] But I couldn't handle that part, I really couldn't. (Inf 4

#### Uncertain subsistence

The informants felt that they were waiting for decisions that were often hard to predict. They experienced great uncertainty about the future, not knowing whether they were going to keep their sickness benefit or not, or whether they were going to be pushed quickly into work, or moved over to disability pension. They felt that they often received vague or incorrect information, and described feeling restricted by fear of doing wrong and losing their allowance.

But I don't know anything. I don't know what is going to happen with my life. Just a thing like I'm lucky to live where I live, because I don't know if they are going to send me back to work all of a sudden [by withdrawing my sickness benefit]. I have no idea what is going to happen with my life. And that is quite mentally stressful. (Inf 5)

The contact with the SIA was described as entailing a constant feeling of being questioned. There was a sense of being treated unfairly, and feeling humiliated when not being believed. Many informants declared that they understood that the SIA had to control the right to sickness benefit, and they stated that overexploitation and fraud made it necessary for social insurance officers to keep up their guard. Despite this understanding, they felt that the officers should see that they themselves were honest. Being questioned by the SIA was described as giving a feeling of not being valued by society.

I felt like... You have an education, you have had a job and struggled and now, the last few years, when you are sick, you get treated like, 'So what? We don't need you anymore.' (Inf 1)

### Content area 2: Interaction with the health care system

In this content area two categories were identified in which the informants described the contact with the health care system as being part of an ongoing process, and being recognized as a person.

#### The importance of being part of an ongoing process

In this category the informants described the importance of feeling involved in an ongoing process of rehabilitation. The experiences in this category varied greatly between informants. Some informants described a continuous rehabilitation process that functioned well.

The good thing is that I have had, how should I say, well, that they have really gone through my body, and done tests. Not just sent me home with a bottle of pills or something. (Inf 7)

Others had experienced years of waiting for one medical investigation after the other, or perceived that they got sick leave notes and analgesics instead of rehabilitation. Some informants described a feeling of putting their lives on hold in the hope of a future solution.

If they had taken all the radiographs in one day and read the results the day after, then they could have done in a week what has now taken more than one-and-a-half-years. If there hadn't been all these waiting times. (Inf 5)

#### The importance of being recognized as a person

In this category the informants described the importance of being recognized as a person. The personal relationship with the treating physician was essential for the experience of the contact with the health care system as a whole. Being respectfully treated and feeling understood were keys to a positive experience. When the physician noticed things that the informant themselves had difficulties explaining or had not yet realized themselves, this was described with great gratitude.

But since I knew that I was suicidal, I thought the doctor should have noticed. I shouldn't have to say that 'I'm thinking of suicide', you simply don't. You just don't do that. And the signals were clear enough, she had to send me to a dietician to get nutritional drinks, since I had stopped eating and such. I mean, I thought those other signals were clear enough. But she refused to put me on sick leave. She said it was work-related problems and that she couldn't help me. (Inf 2)

I can say that I am thankful to my doctor for the rest of my life. I have to say that. Because at the first contact with her she really understood me. Although I didn't know myself what was going on with my health. (Inf 4)

### Content area 3: Recovery and return to work

In this content area four categories emerged, describing essential aspects for recovery and return to work: the possibilities to create conditions for work, the possibilities to create premises for a good life, the link between physical and mental health, and a wish for guidance.

#### The possibilities to create conditions for work

Here the informants expressed a need to try their ability and to be guided in their efforts to return to working life. Being able to adjust the work demands according to their abilities, and being well received in the workplace were important factors for a successful return. Even if the return to work was not successful, the process of trying to was perceived as important to be able to find acceptance.

I work every morning and I rest in the afternoon. And I think that works fine. And at my job they know what's wrong with me, so they don't put all those demands on me, like - you are supposed to cope with stress and do a lot of lifting and such things, and I can't handle that. (Inf 7)

Lack of adjustments was described as leading to failed attempts to return to work.

I was so happy I could manage this job. But when they reorganized the work and all employees had to start doing the cleaning, then - I mean it was huge - everyday there was cleaning - we had to clean the stairs up to the ward and then the whole ward. I mean everything. Imagine failing because of that. (Inf 2)

#### The possibilities to create premises for a good life

The disease was described as a personal loss and the informants had experienced losing normal bodily, cognitive and mental abilities. Being able to adjust the daily routines and learning to handle those limitations was perceived as vital for the quality of life during sick leave. The informants described their recovery and return to work as highly dependent on their own ability to take care of themselves. Support from family and friends sometimes became a necessity for surviving. Many informants experienced economic difficulties and described living with a constant worry about the future. Fear of losing their connection to society by becoming unemployed or having to turn to the social welfare offices was common.

I live in a three-bedroom flat, and I have already moved out of a four-room flat. If I become unemployed I would have to move into a two-room or one-room flat with my two children to manage. It is unbelievably tough. But I don't have family support or any other income to lean on. (Inf 2)

#### The link between physical and mental health

Living with sickness and being on sick leave was depicted as a constant interaction between body and soul. Depression was common among the informants and the degrees of bodily pain experienced and the psychological state of mind were described as strongly dependent on one another. Social isolation led to lost self-esteem and impaired quality of life.

As soon as something happens in my private life, when it gets hard, if the kids have troubles in school or anything - then I can feel it in my body. I can't ignore it any more. (Inf 2)

The isolation, it's a situation where you are completely outside normal life. Working life and friends and also the sense of value [...] Not being able to be active in any way. First of all not being able to be active as a mother, it's terrible. But also not being able to be active in the labour market, or in job situations, when you meet people in a normal life. I thought that was horrible actually. (Inf 13)

#### A wish for guidance

There was a strong and explicit desire for guidance among the informants. They felt the need for more psychological support, but also for support with practical issues concerning their sick leave and the rehabilitation process in general. In some cases individual social insurance officers, social workers or other actors had satisfied this need in periods, but most informants felt disorientation about where to direct this wish.

I would like a 'spider in a web'.... Someone who can help keeping track of things like sick leave, contact with the doctor, medicines, job training. It would have been really useful. Someone who had this task, maybe had a bunch of patients to look after. It would make everything run smoother. (Inf 3)

You are like a reed shaken by the wind. There are different forces acting on you. But it is all about having support and help to see the positive things and what is good, and about ending up with people who have the ability to help. Because you can't expect people on sick leave to be all independent and have access to all their choices. As a person on sick leave you don't feel that way. You are subordinate and you are dependent and afraid of taking steps outwards, for natural reasons you could say. (Inf 13)

## Discussion

### Result discussion

This study shows that people on sick leave consider the interaction with the SIA and health care as an important part of the rehabilitation process, and as affecting recovery and return to work. The experiences of the informants in this study indicate that fragmented interaction can hamper patients' possibilities to take active responsibility for their own rehabilitation process.

Finding the way back to work, from being on sick leave due to musculoskeletal disorders, is often not a matter of 'getting well' or returning to life the way it was before getting ill. It is rather a matter of adjusting to a life with a chronic disease. The informants described going through a process of adjustment in both private and working life. This process of adjustment was interactive, including the possibility to adjust work demands and living conditions, as well as personal and emotional adjustment.

Two major conceptual models of disability have been prominent in the past. The *medical model *focuses on disability as a feature of the person, directly caused by disease, trauma or other health conditions. The *social model *of disability, on the other hand, sees disability as a socially created problem and not an attribute of the individual [21]. The more current *biopsychosocial model *[22] emphasizes the importance of considering the physical, psychological and social aspects of illness and disease. In this model physical disorder, distress and illness behaviour combine to produce disability. The interaction between physical and psychological factors determines the outcome of treatment and the efficacy of the rehabilitation process.

Within the field of rehabilitation the approach to patients has similarly moved from a predominantly medical one to one in which psychological and sociocultural aspects are equally important [23]. This development is reflected in the United Nations definition of the term: 'The term "rehabilitation" refers to a process aimed at enabling persons with disabilities to reach and maintain their optimal physical, sensory, intellectual, psychiatric and/or social functional levels, thus providing them with the tools to change their lives towards a higher level of independence.'[24]

According to this approach, rather than healing the patients, the focus is on helping the patients to cope with their problems [22]. The patient's role, correspondingly, must change from being a passive recipient of treatment to more actively sharing responsibility for his or her own progress [22]. For the sickness absentees to be able to fulfil this more active sharing of responsibility in the rehabilitation process, it is important that they understand which actor is responsible for what in that process. In Sweden the process of rehabilitation involves many actors. The SIA is responsible for the coordination of the rehabilitative activities during sick leave, and ensuring that the needs for rehabilitation are met. The health care system is responsible for the medical rehabilitation, the employment services or the employer for the vocational rehabilitation, and the municipalities for the social measures. This complex network of divided areas of responsibilities might hamper the possibilities for the patients to achieve the necessary overview of the process. The complexity of the system could thus have a disempowering effect for the sickness absentees. In line with this reasoning, the desire for guidance among the informants can be seen as a desire to regain control over one's own rehabilitation. A more coherent process and appropriate professional guidance could consequently have an empowering effect for people on sick leave.

As found in other studies, the informants in this study described confidence and trust as major dimensions of positive encounters [10]. Previous research has also shown that positive experiences of encounters with rehabilitation professionals, in both the health care system and the SIA, affect health outcomes and facilitate return to work [7,9-11,25]. In our study the contact with the SIA was described as containing a constant feeling of being questioned. Simultaneously, there was an understanding of the necessity of controlling the right to sickness benefit. This sheds light on a contradiction between the gatekeeping function and the rehabilitating responsibility that the SIA and treating physicians hold in the Swedish social insurance system. A similar contradiction might risk arising when a stronger emphasis on a medical and 'insurance-focused' approach is implemented in the assessments of the right to sickness benefit. This approach may influence the rehabilitation process towards a more *medical model*, with an increased risk of counterproductive effects, as described by the informants in this study.

### Methodological considerations

In this study we aimed for criterion based, purposive sampling, sometimes called strategic sampling. An ideal strategic sampling would include informants chosen to represent the desired variety of predefined characteristics. Administrative records or databases are sometimes suggested as such sample frames [14]. However, due to patient safety, legal and ethical reasons this was not possible for the present study. Instead we chose gate-keepers strategically, and designed a two-fold way of recruitment using various gatekeepers and advertisements in the recruitment process. By using a combination of the two recruitment processes we hoped to capture a variety of experiences among the informants. A limitation of this method is that the range of characteristics could not be fully determined until after inclusion. However, by checking the variety of characteristics and range of experiences in the final sample, and comparing to the desired variety, our sample covered the predetermined criteria (see Table 2). It could be argued that recruitment through contacts within the SIA and the health care system might lead to an overrepresentation of informants with positive experiences and close contacts with these actors. On the other hand, recruitment through advertisements could lead to an overrepresentation of informants with negative experiences.

**Table 2:** Informant characteristics

Age	
30-39	2

40-49	5

50-65	8

**Sex**	

Women	11

Men	4

**Residence**	

Large community	3

Medium-size community	5

Smaller community	7

**Civil status**	

Living alone	4

Living with children	5

Living with partner	4

Living with partner and children	2

**Country of birth**	

Born in Sweden	12

Born in another European country	3

**Sick leave status**	

Partly back to work	5

Partly unemployed	2

Fully on sick leave	5

Full disability pension	3

**Current basic employment situation**	

Employed	8

Unemployed	3

Student	1

Working in own business	3

**Main diagnosis**	

Rheumatoid arthritis	3

Other rheumatic disease	2

Back or neck problems, slipped disk, lumbago etc	6

Fibromyalgia	2

Stroke	2

Socio-economic and sick leave status data

The interviews were conducted by the first author (JH), who has personal experience of working within both the SIA and the health care system. This could influence the results, although a thorough knowledge of the field is crucial to be able to probe in more depth into experiences that are brought up and also to understand the concepts described by the informants. The different professional and educational backgrounds of the other authors help to broaden the perspective of the analysis and conclusions.

## Conclusion

The informants perceived the interaction with the SIA and the health care system as ranging from being coherent to being fragmented. Coherent interaction was described as facilitating recovery and return to work, while fragmented interaction was described as obstructing this. To create a more coherent rehabilitation process, it is important that the vocational, social, psychological and medical aspects of rehabilitation are coordinated and integrated. To facilitate recovery and return to work, it is crucial to ensure that working conditions are realistic and adapted to the returning person's ability. The rehabilitation process must include support for people on sick leave in creating premises for life and work despite remaining limitations. Working for a more coherent process of rehabilitation and offering professional support to patients might thereby both have an empowering effect and facilitate return to work.

## Competing interests

The authors declare that they have no competing interests.

## Authors' contributions

JH participated in the design of the study, conducted the interviews and the data analysis, and drafted the manuscript. CT and BA participated in the design, analysis and interpretation of the data and critically revised the manuscript. IP participated in the design of the study and critically revised the manuscript. All authors reviewed and approved the final manuscript.

## Pre-publication history

The pre-publication history for this paper can be accessed here:

http://www.biomedcentral.com/1471-2458/11/107/prepub
